# Growth and Non-Thermal Inactivation of *Staphylococcus aureus* in Sliced Dry-Cured Ham in Relation to Water Activity, Packaging Type and Storage Temperature

**DOI:** 10.3390/foods12112199

**Published:** 2023-05-30

**Authors:** Anna Austrich-Comas, Cristina Serra-Castelló, Maria Viella, Pere Gou, Anna Jofré, Sara Bover-Cid

**Affiliations:** 1 Food Safety and Functionality Program, IRTA, Finca Camps i Armet, E-17121 Monells, Spainanna.jofre@irta.cat (A.J.); 2 Food Quality and Technology Program, IRTA, Finca Camps i Armet, E-17121 Monells, Spain; pere.gou@irta.cat

**Keywords:** predictive microbiology, ready-to-eat meat products, shelf-stable food, food safety

## Abstract

Dry-cured ham (DCH) could support the growth of *Staphylococcus aureus* as a halotolerant bacterium, which may compromise the shelf-stability of the product according to the growth/no growth boundary models and the physicochemical parameters of commercial DCH. In the present study, the behavior of *S. aureus* is evaluated in sliced DCH with different water activity (a_w_ 0.861–0.925), packaged under air, vacuum, or modified atmosphere (MAP), and stored at different temperatures (2–25 °C) for up to 1 year. The Logistic and the Weibull models were fitted to data to estimate the primary kinetic parameters for the pathogen Log_10_ increase and Log_10_ reduction, respectively. Then, polynomial models were developed as secondary models following their integration into the primary Weibull model to obtain a global model for each packaging. Growth was observed for samples with the highest a_w_ stored at 20 and 25 °C in air-packaged DCH. For lower a_w_, progressive inactivation of *S. aureus* was observed, being faster at the lowest temperature (15 °C) for air-packaged DCH. In contrast, for vacuum and MAP-packaged DCH, a higher storage temperature resulted in faster inactivation without a significant effect of the product a_w_. The results of this study clearly indicate that the behavior of *S. aureus* is highly dependent on factors such as storage temperature, packaging conditions and product a_w_. The developed models provide a management tool for evaluating the risk associated with DCH and for preventing the development of *S. aureus* by selecting the most appropriate packaging according to a_w_ range and storage temperature.

## 1. Introduction

Dry-cured ham (DCH) has traditionally been considered a safe and microbiologically shelf-stable product because of the combination of hurdles (e.g., low moisture, high salt content and the presence of curing agents) that contribute to inhibiting pathogen growth and/or even promote pathogen inactivation [1,2,3]. However, DCH with high a_w_ has been reported to be associated particularly with commercial pre-packaged sliced products, which may compromise food safety [4]. For instance, according to the survey performed by Hereu [5], 50% of the DCH products sampled from retail showed an a_w_ equal to or higher than 0.92.

Serra-Castelló et al. [6] reported a progressive inactivation of *Listeria monocytogenes* in vacuum-packaged Serrano and Iberian DCH (a_w_ = 0.85–0.91) stored at different temperatures (4 to 25 °C). *Salmonella* viability also decreased on vacuum-packaged DCH stored at 1 to 25 °C [7]. However, compared with other pathogens, *Staphylococcus aureus* is a pathogen of concern for DCH due to its halotolerant nature, which enables it to grow over many adverse conditions, including at low a_w_ (≥0.83) and with high salt concentrations (up to 20%) [8,9,10]. Enterotoxigenic *S. aureus* strains are able to produce staphylococcal enterotoxins (SEs) when the concentration exceeds 5 Log_10_ CFU/g, although SE production requires higher a_w_ (i.e., 0.86) than growth [10,11,12].

The behavior of *S. aureus* has been quite widely studied through challenge tests under laboratory conditions in which food characteristics are mimicked [13,14,15]. However, only a few studies have evaluated the behavior of *S. aureus* on DCHs through challenge testing. Christieans et al. [16] observed no growth at 8 °C for any of the a_w_ studied (0.89–0.96). Conversely, growth was found on DCH samples stored at 20 °C regardless of the a_w_. In another study, *S. aureus* growth on slices of vacuum-packaged DCH was reported at the highest temperature (25 °C); however, no SE was produced after storage for 28 days at 2 and 25 °C [1]. Márta et al. [17] detected SE in Serrano ham with low a_w_ and high salt and fat levels after 5 days when stored aerobically at 23 °C. Unterman and Müller [18] showed that in minced DCH with a_w_ of 0.89, enterotoxin was produced when it was stored at 35 °C for 7 days. These studies tested specific experimental conditions, but none of them were designed to simultaneously cover a wide range of a_w_, storage temperature (from refrigeration to room temperature) and atmosphere compositions (such as air, vacuum and modified atmosphere packaging (MAP) with CO_2_) usually found in commercial DCH. Accordingly, additional studies are needed to be able to draw conclusions regarding the conditions that pose a risk. In this respect, the development and application of predictive models, if available, represent a valuable complementary approach to challenge testing to quantitatively characterize the behavior of pathogens in food [19], to identify either the growth/no growth boundaries, or the growth or the inactivation (survival) kinetics throughout storage [20], which are used to assess the impact of relevant intrinsic and extrinsic factors taking into account the DCH variability [21,22,23]. 

The overall aim of the present study was to evaluate the behavior of *S. aureus* in Spanish dry-cured ham considering intrinsic (a_w_ and pH) and extrinsic factors (storage temperature and packaging conditions) through predictive modeling and challenge testing approaches. First, the physicochemical characteristics of pre-packaged sliced DCH were used as inputs of selected growth/no-growth (G/NG) models to assess the growth probability of *S. aureus* at different temperatures (Study 1). Afterwards, the growth of *S. aureus* was evaluated through challenge testing in DCH slices packaged using different packaging methods (air, vacuum and MAP) and stored at different temperatures (2 to 25 °C) with the subsequent development of three predictive models (Study 2).

## 2. Materials and Methods

### 2.1. Dry-Cured Ham (DCH) Samples

For Study 1, a total of 20 pH and a_w_ historical data provided by a food producer of Spanish DCH, corresponding to different batches and representative of their products, were used. Data representing the physicochemical characteristics of the sliced product (before the final packaging) was used. 

For Study 2, blocks of deboned DCH (pH 5.80 ± 0.06) showing three different levels of a_w_—low, medium and high—were provided by the same food producer and were selected to cover the range of a_w_ variability usually found (ca. 0.860, 0.901, 0.925, respectively). To equalize the value of a_w_ throughout the matrix, DCH blocks were vacuum packaged and stored at 4 °C for 15 days. In this way, the differences in a_w_ value within different sections of a DCH block were lower than 0.028. 

Figure S1 shows a graphical summary of the experimental design of Study 1 and Study 2.

### 2.2. Challenge Test 

#### 2.2.1. Inoculum Preparation

A cocktail of three strains of *S. aureus* was used: CECT976 (SEA producer) and CECT4466 (SED producer), from the Spanish Type Culture Collection, and CTC1008, as a meat isolate from the IRTA culture collection. Each strain was independently grown in Brain Heart Infusion (BHI) broth (Becton Dickinson, Sparks, MD, USA) at 37 °C for 24 h. The cultures were cryopreserved at −80 °C with 20% glycerol until use. Thawed cultures of each strain were mixed at equal concentrations before being inoculated on DCH. 

#### 2.2.2. DCH Inoculation

DCH was aseptically sliced (ca. 20 g/slice) and inoculated in a laminar flow cabinet. The cocktail of the *S. aureus* strains was inoculated on the surface of DCH slices at 0.5% (*v*/*w*) to reach a different final concentration, i.e., from 5 × 10^2^ (to characterize growth) to 2.5 × 10^6^ CFU/g (to characterize inactivation). For air- and vacuum-packaged samples, the inoculum was spread on the surface with a single-use Digralsky spreader. The DCH was packaged in PA/PE bags (oxygen permeability of 50 cm^3^/m^2^/24 h and a low water vapor permeability of 2.8 g/m^2^/24 h; Sistemvac, Estudi Graf SA, Girona, Spain) thermosealed or vacuum packaged (EV-15-2-CD; Tecnotrip, Terrassa, Spain), respectively. Meanwhile, MAP samples were inoculated after packaging (80% N_2_ and 20% CO_2_) with a sterile syringe through a septum to avoid gas leakage.

#### 2.2.3. DCH Storage and Sampling

DCH samples were stored at different temperatures depending on the packaging type: air-packaged samples were kept at 15, 20 and 25 °C; vacuum-packaged samples at 2, 8, 15, 20 and 25 °C; and MAP samples at 2, 8, 15 and 25 °C. Storage time ranged from 1 month for the DCH with the highest a_w_ at the highest temperature up to 1 year for the DCH with the lowest a_w_ at the lower temperature. Sampling points were distributed throughout the storage time. A total of 36 experimental conditions combining a_w_, packaging format and storage temperature were assayed (resulting in 615 data points).

### 2.3. Microbiological and Physicochemical Determinations 

For microbiological analysis, 10 g of sample were transferred into a bag blender Smasher^®^ (bioMérieux, Marcy-l’Étoile, France) and 10-fold diluted and homogenized in physiological saline (0.85% NaCl and 0.1 % Bacto Peptone (Becton Dickinson, Sparks, MD, USA)) for 60 s with a Smasher^TM^ device (bioMérieux Espãna S.A, Madrid, Spain). Serial decimal dilutions were prepared in physiological saline. Enumeration of *S. aureus* was performed on selective and differential chromogenic agar (CHROMagar Staphylococcus, CHROMagar, Paris, France) incubated at 37 °C for 48 h. LAB levels were determined in Man–Rogosa–Sharpe (MRS) agar (Merck, Darmstadt, Germany), incubated at 30 °C for 72 h anaerobically in sealed jars with an AnaeroGen sachet (Oxoid Ltd.)

The a_w_ was measured with an AquaLab^TM^ instrument (Series 3; Decagon Devices Inc., Pullman, WA, USA). The pH was measured with a penetration probe (52-32; Crison Instrument SA, Alella, Spain) connected to a portable pH meter (PH25; Crison Instruments). The detection of SEs was determined according to ISO 19020 [24] by automated immunofluorescence.

The gas concentration of MAP-packaged samples was measured with the gas analyzer PBI Dansensor CheckMate II (Ametek Instrumentos, S.L.U., Barcelona).

### 2.4. Predictive Microbiology Approaches

#### 2.4.1. Growth/No Growth Prediction

For Study 1, predictive models about G/NG boundaries for *S. aureus* were used to identify the pH and a_w_ combinations defining the 10% probability (as a moderately conservative threshold) and predict the growth probability of *S. aureus* associated with the physicochemical characteristics of commercial DCH (Section 2.1). The main features of the selected predictive models used are summarized in Table S1. The G/NG model of Borneman et al. [25] is a logistic regression-based polynomial that determines the probability of *S. aureus* growth on vacuum-packaged RTE meat products at 21 °C with pH and a_w_ as input factors. Polese et al. [14] used a gamma-concept model with pH, a_w_ and temperature as input factors, and the model was tested for a variety of foods stored between 2 to 30 °C. Finally, the model available in the *Sym’Previus* [26] portal predicts the G/NG interface for *S. aureus* depending on a_w_, pH and temperature using a gamma-concept approach and the mean cardinal parameters for the growth of eight *S. aureus* strains. 

#### 2.4.2. Primary Model Fitting

For Study 2, challenge test data were used to estimate kinetic parameters of growth or inactivation by fitting a primary model. For each data point, the Log_10_ change in the concentration in relation to the initial inoculum concentration (e.g., Log_10_ increase or Log_10_ reduction) was calculated as Log_10_ (*N*/*N*_0_), *N* is the concentration (CFU/g) at the sampling time and *N*_0_ is the initial concentration (CFU/g) after inoculation of DCH samples. 

For conditions supporting the growth of *S. aureus*, the Logistic model (Equation (1)) [27] was used to estimate the growth kinetic parameters.

(1) $$\begin{array}{l} \mathrm{For}\;t<\lambda ,\;Log\left(\frac{N_{t}}{N_{0}}\right)=0\\ \mathrm{For}\;t\geq \lambda ,\;Log\left(\frac{N_{t}}{N_{0}}\right)=Log\left(\frac{MGP}{1+\left(MGP-1\right)*\left(exp\left(-{µ}_{max}*\left(t-\lambda \right)\right)\right)}\right) \end{array}$$

where *N*_0_ is the concentration of the pathogen (CFU/g) at time zero; *N_t_* is the concentration of the pathogen (CFU/g) at time *t*; *MGP* is the maximum growth potential as the ratio *N_max_*/*N*_0_ (*N_max_* is the maximum population density, CFU/g); *λ* is the lag time (h); μ*_max_* is the maximum specific growth rate (ln/h); and *t* is the storage time (h).

For conditions causing the inactivation of *S. aureus*, the Weibull model (Equation (2)) [6] was used to estimate the inactivation kinetic parameters.

(2) $$Log\left(\frac{N}{N_{0}}\right)=-{\left(\frac{t}{\delta }\right)}^{p}$$

where Log_10_ (*N*/*N*_0_) is the inactivation in Log_10_ reduction (Log_10_ units) at a given time (*t*) of the storage, being equal to 0 at storage time 0; *t* is the storage time (h); *δ* is the time (h) for the first Log_10_ reduction and *p* is the shape of the inactivation curve. 

Model fitting was performed with the *nls2* package of R software [28]. In addition to the standard error of the estimates, to evaluate the goodness of fit, the Root Mean Square Error (RMSE) values were calculated.

#### 2.4.3. Secondary and Global Model Fitting 

To evaluate the effect of storage temperature and DCH’s a_w_ on the inactivation kinetics parameters (*δ*, *p*), a secondary model was developed based on a second-order polynomial equation (Equation (3)) for each packaging condition.

y = *a* + *b*·X + *c*·X^2^ (3)

where y is the dependent variable, i.e., the primary kinetic parameter (e.g., *δ*), X is the independent variable, i.e., the environmental factor (e.g., temperature), and *a*, *b* and *c* are the model coefficients to be estimated.

Different parameter transformations (including square root and Log_10_) were assessed. The stepwise linear regression was applied throughout the *step* function of the R software [28] to obtain the polynomial models with only significant parameters according to the parsimony principle. In addition to the standard error of the estimates, the goodness of fit was assessed in terms of RMSE and the adjusted coefficient of determination (R^2^_adj_). 

In addition to the classical two-step modeling (primary and secondary model fitting), the one-step modeling approach was applied by integrating the secondary polynomial model for *δ* into the primary Weibull model. The global model was fitted to the whole dataset to obtain a global model with re-adjusted coefficients for each type of packaging [29,30]. Estimation of the model parameters with the standard error was carried out using *nls2* package of R software [28]. The goodness of fit of the global model was assessed on the basis of RMSE. The F-test (Equation (4)) was used to evaluate the statistical differences (*p* < 0.05) between the models developed to characterize the behavior of *S. aureus* in air, vacuum, and MAP conditions [31].

(4) F=(RSSNH−RSSAH)(dfNH−dfAH)RSSAH−dfAH

where *RSS_NH_* and *df_NH_* are the Residual Sum of Squares and the degrees of freedom (number of points minus the number of parameters of the model), respectively, of the global model common to all types of packages (null hypothesis), and *RSS_AH_* and *df_AH_* are the Residual Sum of Squares and the number of degrees of freedom, respectively, of the global model with specific parameter coefficients for each type of package (alternative hypothesis). 

Moreover, due to the statistical correlation between *δ* and *p* parameters [32], the F-test was applied to test the statistical significance of the effect of storage temperature and the DCH’s a_w_ on the shape of the inactivation curve of *S. aureus*. The global model with a fixed *p* value independent of environmental conditions (null hypothesis) was compared with the global model with a polynomial model describing the effect of environmental conditions on the *p* parameter (alternative hypothesis). 

#### 2.4.4. Model Validation

In order to evaluate the predictive performance of the developed model, the Acceptable Simulated Zone (ASZ) was applied [33]. Independent data were obtained from three published articles dealing with the behavior of *S. aureus* in dry-cured ham, with a total of 80 sampling points: 7 for air-packaged DCH, 24 for vacuum-packaged DCH, and 49 for MAP-packaged DCH. Log_10_ count data over time were extracted from published scientific literature using WebPlotDigitizer v.4.4 software. The observed and predicted Log_10_ reduction data during the storage time were compared. The predictive performance of the model was considered acceptable when at least 70% of the independent data were inside the ASZ ± 0.5 Log_10_.

## 3. Results

### 3.1. Characteristics of Commercial DCH and the Associated Probability of S. aureus Growth (Study 1)

**Figure 1:**
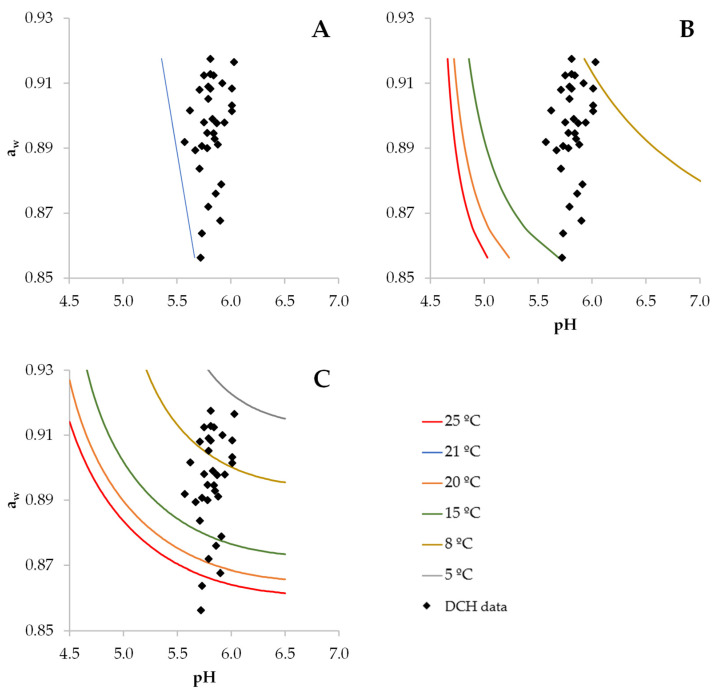
Distribution of pH and a_w_ values of commercial dry-cured ham (DCH, diamond dots) and pH–a_w_ boundaries for the growth probability of 10% for *S. aureus* at different temperatures according to the predictive models (lines) available in Borneman et al. [25] (**A**), Polese et al. [14] (**B**) and the Sym’Previus portal [26] (**C**).

The distribution of physicochemical characteristics (a_w_ and pH) of commercial vacuum-packaged DCH is shown in Figure 1, with the prediction of the growth boundaries according to the predictive models available for *S. aureus*. Despite a 50% probability of growth being frequently used to assess the G/NG boundary, in the present study, a growth probability of 10% was used as a conservative reference boundary. Although the variability of the pH was rather limited (within 5.5. to 6.0), the values of a_w_ were scattered within a range from 0.85 to 0.92, with a considerable proportion (82%) of samples at above 0.88, the minimum a_w_ for growth reported for anaerobic conditions when the other factors (pH and temperature) were optimal for growth [34]. In fact, according to the models of Borneman et al. [25] and Polese et al. [14], for all the observed DCH characteristics, a growth probability higher than 10% was predicted at temperatures above 15 °C, while only 15% of samples would not support growth (probability below 10%) at these temperatures according to the model of the Sym’Previus portal [26]. Only when storage temperature decreased to below 8 °C for Polese et al. model [14] and to below 5 °C for Sym’Previus model [26] did the growth probability fall below 10% for almost all samples, indicating that refrigeration storage would be needed to control the growth of *S. aureus* in DCH during the shelf life.

However, these predictive models were not specifically developed for DCH, and do not take into consideration the effect of relevant factors related to the specific characteristics of the product (i.e., lactic acid concentration, lactic acid bacteria) and packaging (e.g., oxygen reduction of vacuum packaging and CO_2_ concentration of MAP), which may contribute to further inhibiting the growth of *S. aureus*. Therefore, product-specific studies were required.

### 3.2. Behavior of S. aureus on Sliced DCH Stored under Different Conditions (Challenge Test Experiment, Study 2)

**Figure 2:**
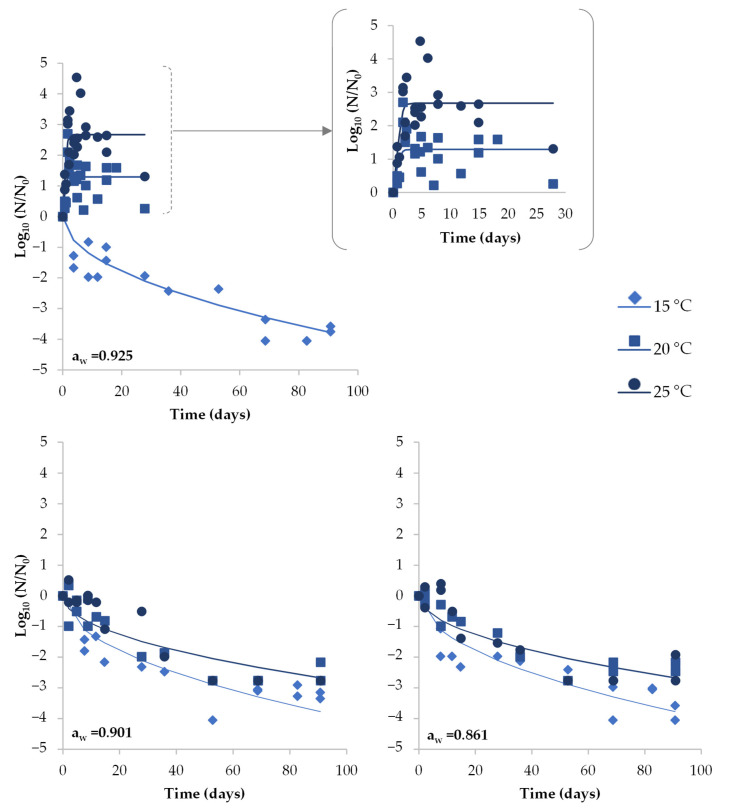
Behavior of *S. aureus* in sliced DCH with different a_w_ (0.861, 0.901 and 0.925) when air packaged and stored at different temperatures (15, 20 and 25 °C). Dots represent the observed *S. aureus* values (Log_10_ *N*/*N*_0_). Lines show the fit of the global model.

**Figure 3:**
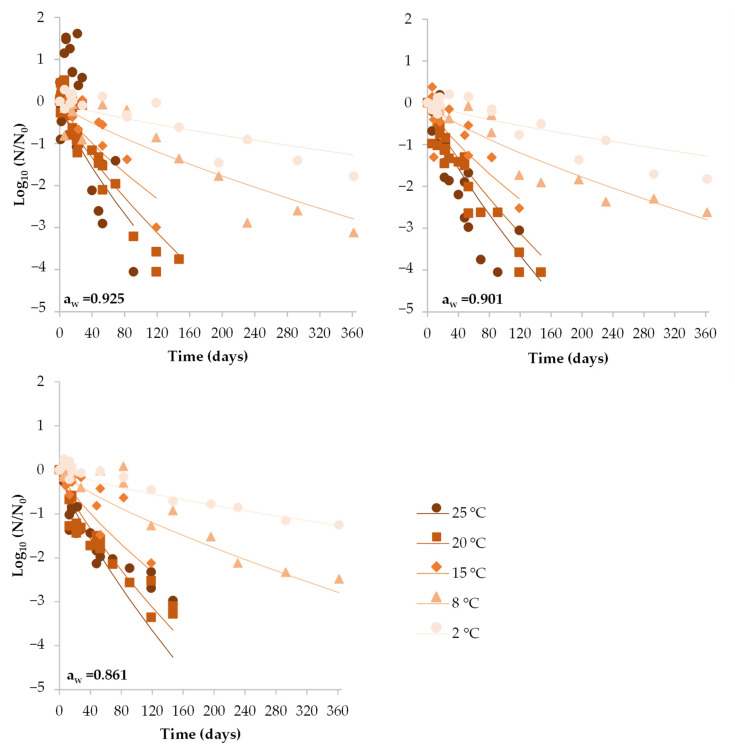
Behavior of *S. aureus* in DCH sliced with different a_w_ (0.861, 0.901 and 0.925) when vacuum packaged and stored under different storage temperatures (2, 8, 15, 20 and 25 °C). Dots represent the observed *S. aureus* values (Log_10_ *N*/*N*_0_). Lines show the fit of the global model.

Growth of *S. aureus* was observed in three out of the 36 trials, which corresponded to those where DCH had the highest a_w_ (0.925) stored in air at temperatures ≥20 °C and under vacuum at 25 °C (Figure 2 and Figure 3). The growth kinetic parameters estimated for each trial are shown in Table 1, including the growth rate (μ*_max_*) and the maximum growth potential (MGP). No lag time was observed. In air-packaged DCH, *S. aureus* increased by up to 2.7 and 4.54 Log_10_ units after 1.7 and 4.7 days of storage at 20 and 25 °C, respectively. At 25 °C, the growth rate was slightly higher compared to the growth at 20 °C. However, due to the high variability in Log_10_ increase data (especially at 20 °C), growth rates were not statistically different. Under vacuum, a slight increase in *S. aureus* (1.62 Log_10_ units in 22 days) was observed during the early stages of storage at 25 °C in the DCH with the highest a_w_. Afterwards, the pathogen started to die off, and growth kinetic parameters could not be estimated.

Under the rest of assessed conditions, inactivation of *S. aureus* was observed, and the kinetic parameters estimated with the Weibull model fit, i.e., the time for the first Log_10_ reduction (*δ)* and the shape of the inactivation curve (*p*) were obtained (Table 1).

In air-packaged DCH with medium a_w_ (0.902) and low a_w_ (0.861), *S. aureus* concentration had decreased by 2.5 Log_10_ units after 91 days at 20 and 25 °C, while higher inactivation occurred at 15 °C, with reductions of 3.66, 3.25 and 3.81 Log_10_ in DCH with high, medium and low a_w_ after 91 days, respectively (Figure 2). Higher *δ* values were found with increasing storage temperature (Table 1), although the kinetic curve at 20 °C was very similar to that at 25 °C (Figure 2). Conversely, similar *δ* values were observed for DCH with different a_w_ values for each storage temperature. Regarding the shape of the inactivation curve, the fit of the Weibull model resulted in *p* values below 1 for all the studied conditions, regardless of the storage temperature and DCH a_w_, indicating a more pronounced inactivation of *S. aureus* at the early stages of the storage followed by a sort of a resistance tail showing a slower inactivation.

Under vacuum conditions, the progressive inactivation of *S. aureus* after a slight increase during the first 22 days in DCH with the highest a_w_ stored at 25 °C resulted in an overall 4.05 Log_10_ reduction after 91 days. For the rest of the temperatures studied, *S. aureus* was unable to grow at all (Figure 3). Instead, a progressive reduction was observed, which was dependent on the storage temperature but not on the product a_w_. Contrary to air-packaged DCH, for vacuum-packaged the time for the first Log_10_ reduction (*δ* value) decreased as the temperature increased from 2 to 25 °C (Table 1).

**Figure 4:**
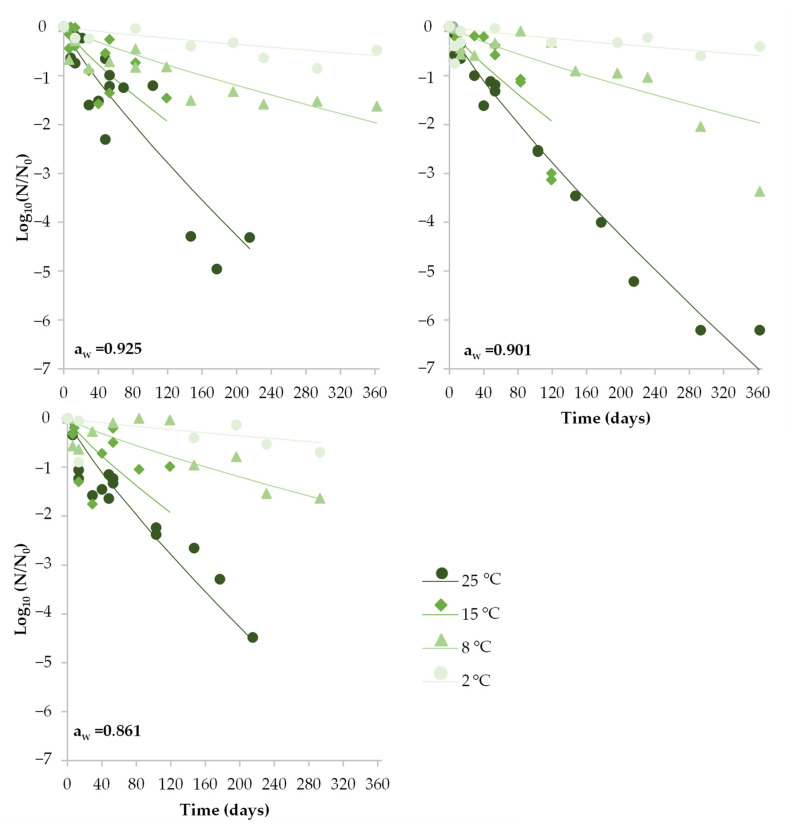
Behavior of *S. aureus* in sliced DCH with different a_w_ (0.861, 0.901 and 0.925) when MAP packaged and stored under different storage temperatures (2, 8, 15 and 25 °C). Symbols represent the observed *S. aureus* inactivation (Log_10_ *N*/*N*_0_). Lines show the fit of the global model.

Storage under MAP promoted the loss of viability of *S. aureus* in all combinations of a_w_ and temperature tested from the beginning of the storage. As in other packaging types, the a_w_ of DCH had no relevant effect on inactivation (Figure 4), with similar *δ* values for DCH with different a_w_ for each storage temperature. The extent of the inactivation in MAP tended to be lower than under vacuum-packaged conditions. Moreover, at the lowest temperature (2 °C), no microbiologically relevant inactivation (less than 1 Log_10_) occurred in DCH with medium and high a_w_ during the 365 days of storage, making the estimates of *δ* values higher than the studied storage time.

### 3.3. Physicochemical Determinations and Lactic Acid Bacteria Counts

The physicochemical characteristics were measured throughout the study. As all DCH samples were packaged with impermeable bags, values of a_w_ of DCH did not change during the storage at any temperature. The values of pH changed slightly depending on the packaging type and temperature or the a_w_ values (Table S2). The behavior of LAB levels during the storage time depended on the a_w_ of the DCH, the packaging type and the storage temperature (Table S3). Under air packaging, LAB was not able to grow at lower a_w_, irrespective of the temperature; LAB was able to grow only in the DCH with medium and high a_w_ at all three temperatures tested, with a slight pH reduction (i.e., a 4.39 Log_10_ increase in LAB was associated with a 0.17 pH decrease in DCH with high a_w_ stored at 25 °C). Under vacuum packaging gand MAP, a similar trend was observed. At lower temperatures, no LAB growth was observed. Additionally, at higher temperatures (>15 °C), in general, LAB was able to grow, and a reduction in pH was observed, especially at 40 days of storage.

No differences in the gas composition on MAP-packaged DCH were detected over the course of the storage time.

The potential occurrence of staphylococcus enterotoxin (SE) was analyzed in DCH samples where the *S. aureus* grew at the maximum concentration, which corresponded to air-packaged DCH with higher a_w_ stored at 25 °C (6.38 and 5.87 Log_10_ CFU/g). No SEs were detected.

### 3.4. Secondary and Global Modeling

**Figure 5:**
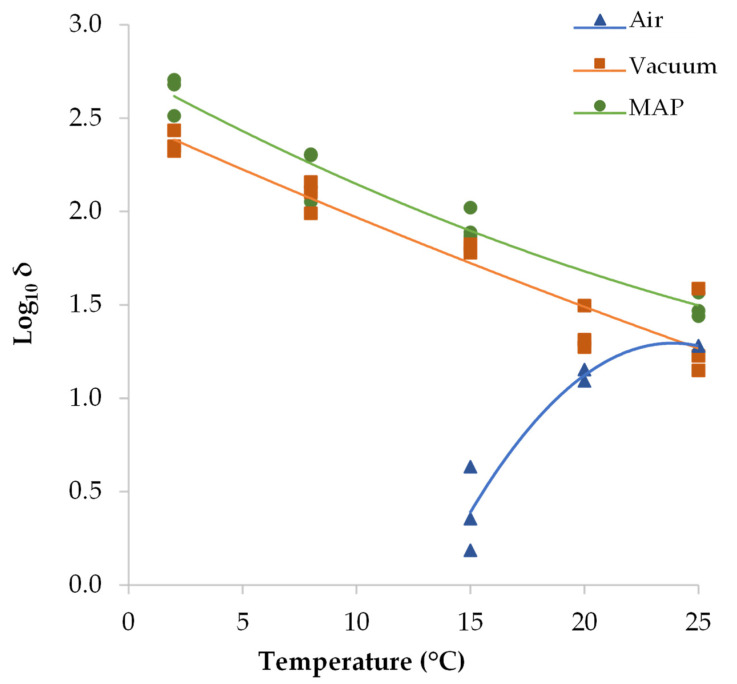
Time for the first Log_10_ reduction (*δ* value) of *S. aureus* in dry-cured ham (DCH) as a function of storage temperature for different packaging conditions (air, vacuum and MAP). Dots are the values estimated with the primary inactivation model (Weibull) and lines correspond the fit of the second-order polynomial model to Log_10_ transformed *δ* values.

To describe the effect of storage temperature and a_w_ on the inactivation kinetics of *S. aureus* through a polynomial model, different transformations of the parameters (*δ* and *p*) were assessed, and the Log_10_ transformation of *δ* values provided the best results. Table 2 gathers the coefficients of the polynomial model and Figure 5 shows the Log_10_ *δ* values as a function of temperature for the three different packaging conditions. Only the temperature, and not the a_w_, was found to significantly affect *δ* values in each packaging type. The effect of storage temperature on the time for the first Log_10_ reduction (*δ* values) in air-packaged DCH (*δ* decreased as temperature decreased) was the opposite of that in packaging systems without oxygen, i.e., vacuum packaging and MAP (*δ* decreased as temperature increased).

Regarding the *p* parameter, no clear relationship could be established with either storage temperature or the product a_w_, as indicated by the lack of fit of the polynomial model, not even with the different parameter transformations. Therefore, a fixed parameter corresponding to the mean *p* value for all the tested temperatures for each packaging condition (air, vacuum and MAP) was assumed.

A global (one-step) model integrating the secondary model for *δ* values into the Weibull primary model with a fixed *p* value for each packaging type was fitted to Log_10_ reduction data. The re-adjusted parameter estimates are shown in Table 3 (see the final equation in Table S4). All were statistically significant (*p* < 0.05). Despite the similar trend, the global model for vacuum and MAP were significantly different according to the F-test (F = 16.61, *p* > 0.05); therefore, specific model coefficients for each type of packaging are needed to predict the inactivation of *S. aureus* during the storage of DCH.

### 3.5. Predictive Performance of the Model

**Figure 6:**
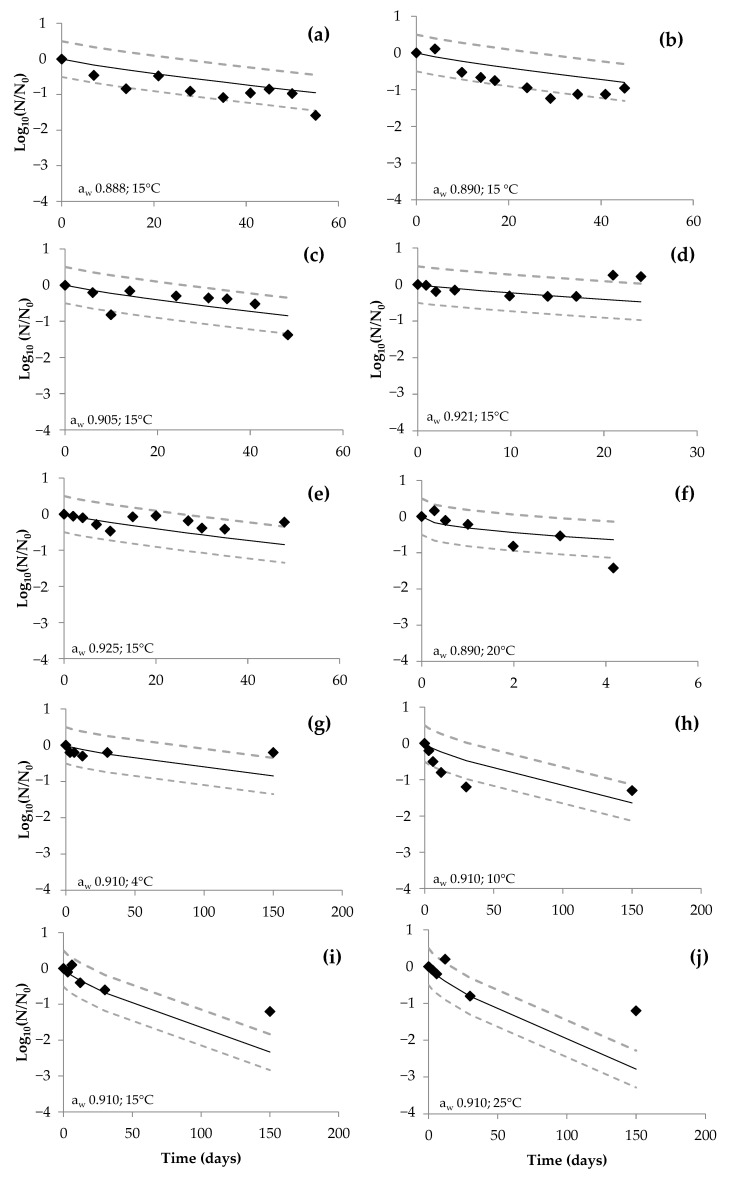
Observed Log_10_ reduction values (dot circles) and acceptable prediction zone −0.5 (fail—safe) to +0.5 (fail—dangerous) from different studies with respect to time (days); (**a**–**e**) data from Christieans et al. [16] in DCH with a_w_ values of 0.888 (**a**), 0.890 (**b**), 0.905 (**c**), 0.921 (**d**), 0.925 (**e**); (**f**) data from Untermann and Müller [18]; (**g**–**j**) data from Iacumin et al. [35] stored at 4 °C (**g**), 10 ºC (**h**), 15 °C (**i**) and 25 °C (**j**).

The predictive performance of the obtained global models (Table S5) was assessed using independent data obtained from three scientific articles dealing with *S. aureus* behavior during the storage of DCH for each packaging condition (i.e., Christieans et al. [16]; Untermann and Müller [18] and Iacumin et al. [35]), with a total of 80 data points. Considering an Acceptable Simulation Zone (ASZ) of ±0.5 Log_10_ units around the model predictions, 85.7% (6/7), 75% (18/24) and 83.67% (41/49) of agreement between the observed values and the model predictions were found for air, vacuum and MAP packaging, respectively. These results indicate a good predictive performance of the developed model in the wide range of a_w_ and temperature conditions assayed (Figure 6, Table S4).

## 4. Discussion

According to available predictive models, the physicochemical characteristics of most commercial DCH would support the growth of *S. aureus* with a probability higher than 10% when stored at >15 °C. The challenge test results confirmed the ability of *S. aureus* to grow on DCH, with the highest a_w_ tested (0.925) being obtained when stored at ≥20 °C under air conditions. These results are in agreement with those reported in Untermann and Müller [18] regarding the growth of *S. aureus* in DCH stored aerobically at temperature ≥20 °C, with an a_w_ of 0.918 and a pH of 5.60–6.07. However, staphylococcal enterotoxin (SE) was not detected in any of the DCH in which the maximum growth was observed (up to 10^5^ CFU/g). Several studies have reported the production of SEs in different food matrixes when *S. aureus* reached 10^5^–10^6^ CFU/g [36,37,38], while others have reported no detection of SEs even at pathogen levels of up to 10^9^–10^10^ CFU/g [39].

In DCH of medium and low a_w_, under storage temperatures equal to or below 15 °C or when DCH was packaged without oxygen (i.e., vacuum and MAP), the viability of the pathogen was compromised. It is known that *S. aureus* grows better under air conditions, as it is a poorly competitive pathogen compared with other microorganisms, in particular LAB, which usually exerts a growth-inhibitory effect on *S. aureus* during meat fermentation processes associated with acidification and the production of antimicrobial substances [40,41]. In general, greater *S. aureus* inhibition is observed with higher LAB concentration and lower pH [42]. Although DCH does not go through a fermentation process, the DCHs studied in this work (i.e., medium- and high-a_w_ products) supported the growth of LAB, which grew faster and reached higher concentrations in DCH with oxygen-reduced packaging (i.e., vacuum packaging and, particularly, MAP) compared with air-packaged DCH. This LAB growth explains, at least partially, the small amount and lack of growth of *S. aureus* observed on DCH when vacuum packaged, as has been reported for raw beef [37]. Moreover, the addition of CO_2_ in MAP-packaged DCH may have favored the selective growth of LAB that, in addition to the antimicrobial effect of CO_2_, could promote the greater inactivation of *S. aureus* behavior.

The progressive loss of viability of microorganisms in harsh conditions occurring in shelf-stable foods such as DCH has been related to the metabolic exhaustion phenomenon associated with antimicrobial hurdles [6,43]. Due to this phenomenon, microorganisms tend to die, and their rate of death is faster when shelf-stability conditions approach the limits of growth [3], which in the present study would be the storage of DCH with the highest a_w_ at the highest temperature when vacuum or MAP packaged. Similar behavior has been reported for *L. monocytogenes* in vacuum-packaged DCH (a_w_ = 0.85–0.91) stored at different temperatures (4 to 25 °C) [6]. On the contrary, in DCH stored under air conditions, the inactivation of *S. aureus* at 15 °C was significantly enhanced compared to that observed at 20 and 25 °C. To the best of the authors’ knowledge, there are no previous studies dealing with the effect of temperature on the non-thermal inactivation of *S. aureus* in DCH that compare aerobic and anaerobic environments. However, in the study of Ha et al. [44], the inactivation of *S. aureus* inoculated on beef jerky (a_w_ = 0.81) followed a similar trend during aerobic storage, and *δ* values at 20 and 25 °C were very similar, and were longer than that at 10 °C, confirming the higher inactivation at lower temperature. Therefore, the non-thermal inactivation seems to be affected by different mechanisms when oxygen is present compared with the anaerobic conditions occurring in vacuum and MAP. In any case, the a_w_ of the DCH did not have a significant effect on the *S. aureus* behavior for any of packaging types, in contrast to the reported behavior of *L. monocytogenes* in DCH [6] and *Salmonella* in dry-fermented sausages [43]. The halotolerance of *S. aureus* may explain the lack of the effect of decreasing the a_w_ of the DCH, at least within the range studied in the present study.

The modeling approach provided predictive models for the three packaging types with a satisfactory performance when assessed with independent data, which supports their suitability for predictive and simulation purposes. This fact provides a management tool for evaluating the risk associated with DCH and to prevent the development of *S. aureus* by selecting the most appropriate packaging according to a_w_ range and storage temperature.

## 5. Conclusions

DCH can support the growth of *S. aureus* at the a_w_ values found in commercial products (ca. 0.92) when stored at room temperature under aerobic conditions, although no staphylococcal enterotoxin was detected. Storage temperature ≤15 °C and reduced-oxygen packaging (i.e., vacuum packaging and, particularly, MAP) inhibits *S. aureus* growth and promotes its inactivation. The product a_w_ does not affect the survival of *S. aureus* on DCH, while the storage temperature has contrary effects in aerobic (higher inactivation at lower storage temperature) and anaerobic packaging (higher inactivation at higher storage temperature), suggesting the involvement of different mechanisms depending on the presence of oxygen in the environment. The predictive models developed are useful tools for stakeholders (e.g., risk assessors, food business operators, competent authority, etc.) for assessing and quantifying the behavior of *S. aureus* on sliced DCH commercialized in different packaging types as a function of storage temperature.

## Figures and Tables

**Table 1:** Estimated kinetic parameters (for the inactivation or growth) resulting from fitting the primary models to data obtained for each challenge test of *S. aureus* on DCH with different a_w_ contents and types of packaging when stored at various storage temperatures.

Experimental Conditions			Kinetic Parameters *^a^*				Goodness of Fit *^b^*	
Packaging	a_w_	Temperature (°C)	Inactivation Weibull Model		Growth Logistic Model		n	RMSE
			*δ* (Days)	*p*	µ*_max_* (ln/h)	MGP (Log_10_)		
Air	0.861	15	2.26 ± 1.43	0.34 ± 0.07	-	-	15	0.446
		20	12.38 ± 3.64	0.48 ± 0.09	-	-	15	0.369
		25	19.01 ± 6.15	0.68 ± 0.17	-	-	15	0.553
	0.901	15	1.53 ± 1.03	0.30 ± 0.06	-	-	15	0.389
		20	14.21 ± 3.88	0.58 ± 0.10	-	-	17	0.453
		25	19.11 ± 4.30	0.80 ± 0.13	-	-	17	0.435
	0.925	15	4.29 ± 1.56	0.44 ± 0.06	-	-	18	0.465
		20	-	-	0.12 ± 0.06	1.29 ± 0.14	25	0.584
		25	-	-	0.17 ± 0.04	2.67 ± 0.19	24	0.737
Vacuum	0.861	2	271.65 ± 15.04	1.11 ± 0.15	-	-	18	0.135
		8	143.59 ± 14.01	1.11 ± 0.15	-	-	18	0.271
		15	66.56 ± 8.23	1.07 ± 0.25	-	-	16	0.325
		20	18.79 ± 2.25	0.58 ± 0.04	-	-	24	0.264
		25	16.81 ± 2.29	0.50 ± 0.04	-	-	24	0.255
	0.901	2	210.43 ± 14.81	1.27 ± 0.20	-	-	18	0.219
		8	97.69 ± 13.21	0.82 ± 0.11	-	-	18	0.305
		15	60.01 ± 7.60	1.24 ± 0.29	-	-	17	0.395
		20	20.57 ± 2.28	0.73 ± 0.05	-	-	24	0.316
		25	14.06 ± 3.10	0.64 ± 0.08	-	-	23	0.551
	0.925	2	223.31 ± 16.41	1.30 ± 0.23	-	-	18	0.232
		8	126.57 ± 18.30	1.11 ± 0.19	-	-	18	0.393
		15	64.04 ± 3.77	1.74 ± 0.19	-	-	17	0.209
		20	31.25 ± 2.98	0.95 ± 0.07	-	-	34	0.347
		25	38.55 ± 6.83	1.70 ± 0.45	-	-	33	0.936
MAP	0.861	2	324.91 ± 68.51	2.66 ± 2.12	-	-	14	0.332
		8	199.73 ± 18.99	1.66 ± 0.47	-	-	15	0.343
		15	104.76 ± 80.30	0.42 ± 0.28	-	-	14	0.516
		25	27.45 ± 5.12	0.67 ± 0.08	-	-	17	0.386
	0.901	2	506.40 ± 252.13	1.80 ± 1.56	-	-	16	0.301
		8	202.56 ± 14.40	2.04 ± 0.30	-	-	16	0.292
		15	77.35 ± 2.66	2.61 ± 0.22	-	-	16	0.163
		25	29.39 ± 3.32	0.77 ± 0.04	-	-	18	0.310
	0.925	2	478.37 ± 126.42	1.16 ± 0.44	-	-	16	0.193
		8	112.95 ± 18.42	0.47 ± 0.09	-	-	16	0.247
		15	73.63 ± 14.38	0.88 ± 0.32	-	-	19	0.411
		25	36.73 ± 7.60	0.90 ± 0.13	-	-	20	0.622

*^a^*: Parameter estimate ± standard error: “-” not applicable *^b^*: n number of data points used for model fitting; RMSE: root mean squared error (Log_10_ units).

**Table 2:** Coefficients of polynomial models describing the effect of storage temperature (T, °C) on Log_10_ *δ*.

Packaging	Coefficients *^a^*			Goodness of Fit *^b^*		
	*a* (Intercept)	*b* (T)	*c* (T^2^)	n	RMSE	$R_{adj}^{2}$
Air	−5.254 ± 1.191	0.549 ± 0.125	−0.0115 ± 0.0031	7	0.261	0.675
Vacuum	2.493 ± 0.031	−0.055 ± 0.006	0.0002 ± 0.0002	15	0.141	0.895
MAP	2.752 ± 0.028	−0.068 ± 0.005	0.0007 ± 0.0002	12	0.103	0.947

*^a^* Parameter estimate ± standard error. *^b^* n: number of data points; RMSE: root mean squared error (Log_10_ units).

**Table 3:** Coefficients of the global (one-step) model about the effect of storage temperature (T, °C) on *S. aureus* inactivation in DCH, integrating the secondary polynomial model into the primary Weibull model for each packaging type.

Packaging	Coefficients of the Polynomial Models *^a^*				Goodness of Fit *^b^*	
	*δ*			*p*	n	RMSE
	*a* (Intercept)	*b* (T)	*c* (T^2^)			
Air	−1.848 ± 0.844	0.263 ± 0.087	−0.006 ± 0.002	0.495 ± 0.034	112	0.498
Vacuum	2.597 ± 0.086	−0.090 ± 0.013	0.002 ± 0.000	0.768 ± 0.050	311	0.441
MAP	3.088 ± 0.135	−0.133 ± 0.019	0.003 ± 0.001	0.838 ± 0.048	193	0.436

*^a^* Parameter estimate ± standard error. *^b^* n: number of data; RMSE: root mean squared error (Log_10_ units).

## Data Availability

Data available on request.

## Supplementary Materials

The following supporting information can be downloaded at: https://www.mdpi.com/article/10.3390/foods12112199/s1, Figure S1: Graphical scheme of the experimental design of Study 1 and Study 2. Table S1: Main features of predictive models for growth/no growth (G/NG) boundaries of *S. aureus* used in Study 1; Table S2: pH values of DCH with different a_w_ content and type of packaging stored at various storage temperatures; Table S3: Lactic acid bacteria counts (Log_10_ CFU/g) measured in DCH with different a_w_ content and type of packaging stored at various storage temperatures; Table S4. Main features of predictive models for growth/no growth (G/NG) boundaries of S. aureus used in Study 1. Table S5: Comparison of observed and predicted *S. aureus* inactivation in sliced DCH.
