# Supplementary material for: Growth and Non-Thermal Inactivation of Staphylococcus aureus in Sliced Dry-Cured Ham in Relation to Water Activity, Packaging Type and Storage Temperature
Source: Foods. 2023 May 30;12(11):2199. doi: 10.3390/foods12112199 (PMC10252274; doi:10.3390/foods12112199)
Supplement: Supplementary file 1 [file foods-12-02199-s001.zip › Table S1.pdf]

Table S1. Main features of predictive models for growth/no growth (G/NG) boundaries of *S. aureus* used in Study 1.

Reference	Modelling approach adopted	Independent variables [range of applicability]		Food matrix of application
		Intrinsic	Extrinsic	
**Borneman et al.** [25]	Logistic regression polynomial	pH [4.0 – 7.0]<br>a_w [0.68-0.98]	Temperature [21ºC]<br>Time [28 days]	Vacuum-packaged meat products (dry and semi-dry fermented sausages, jerky, etc.)
**Polese et al.**[14]	Gamma model[a]	pH [4.0 to 7.0]<br>a_w [0.88 to 0.99]	Temperature [0-30 ºC]	Various
**Sym'Previus portal** [26]	Gamma model	-	-	-

[a]: preservatives such as organic acids are also included in the model structure, but they are not used in the present study.
