# Supplementary material for: Growth and Non-Thermal Inactivation of Staphylococcus aureus in Sliced Dry-Cured Ham in Relation to Water Activity, Packaging Type and Storage Temperature
Source: Foods. 2023 May 30;12(11):2199. doi: 10.3390/foods12112199 (PMC10252274; doi:10.3390/foods12112199)
Supplement: Supplementary file 1 [file foods-12-02199-s001.zip › Table S2.pdf]

Table S2. pH values of DCH with different a_w content and type of packaging stored at various storage temperatures.

Packaging	a_w	T°C	Time (days)									
			0	5	12	16	28	40	147	196	231	293
**Air**	**0.861**	**15**	5.69±0.00	5.74±0.01	-	-	5.73	-	-	-	-	-
		**20**	5.69±0.00	5.69±0.02	5.71	-	5.62	-	-	-	-	-
		**25**	5.69±0.00	5.69±0.01	5.79	-	5.67	-	-	-	-	-
	**0.901**	**15**	5.71±0.00	5.65±0.04	-	-	5.69	-	-	-	-	-
		**20**	5.71±0.00	-	5.68	-	-	-	-	-	-	-
		**25**	5.71±0.00	-	5.54	-	5.60	-	-	-	-	-
	**0.925**	**15**	5.78±0.00	-	5.79	-	5.73	-	-	-	-	-
		**20**	5.78±0.00	5.89±0.03	5.81	-	5.71	-	-	-	-	-
		**25**	5.78±0.00	5.82±0.01	5.69	-	5.74	-	-	-	-	-
**Vacuum**	**0.861**	**2**	5.69±0.00	-	5.75±0.01	5.63±0.02	5.65	-	-	5.88	5.70	5.75±0.00
		**8**	5.69±0.00	-	5.89±0.10	5.81±0.03	5.78	-	-	5.81	5.85	5.93±0.00
		**15**	5.69±0.00	-	5.82±0.02	5.78±0.01	5.73	-	-	5.61	5.66	5.76±0.00
		**20**	5.69±0.00	-	5.75±0.01	5.68±0.01	5.55	-	-			
		**25**	5.69±0.00	-	5.62±0.00	5.43±0.04	5.48	-	-			
	**0.901**	**2**	5.71±0.00	-	5.82±0.06	5.77±0.00	5.68	-	-	5.73	5.77	5.85±0.00
		**8**	5.71±0.00	-	5.92±0.04	5.79±0.02	5.76	-	-	5.82	5.78	5.84±0.00
		**15**	5.71±0.00	-	5.76±0.05	5.74±0.01	5.71	-	-	-	5.66	5.88±0.00
		**20**	5.71±0.00	-	5.66±0.04	5.59±0.04	5.46	5.48	5.64±0.14	-	-	-
		**25**	5.71±0.00	-	5.59±0.00	5.44±0.04	5.57	5.55	5.88	-	-	-
	**0.925**	**2**	5.78±0.00	-	5.90±0.07	5.86±0.06	5.84	-	-	5.94	5.91	5.84±0.00
		**8**	5.78±0.00	-	5.80±0.03	5.84±0.01	5.75	-	-	5.70	5.91	5.74±0.00
		**15**	5.78±0.00	-	5.76±0.05	5.97±0.01	5.70	-	-	-	6.05	5.91±0.00
		**20**	5.78±0.00	-	5.70±0.02	5.55±0.05	5.51	5.63	5.95	-	-	-
		**25**	5.78±0.00	-	5.64±0.06	5.58±0.00	5.568	5.71	-	-	-	-
**MAP**	**0.861**	**2**	5.69±0.00	-	5.89±0.02	-	5.71	-	-	5.65	5.81	5.62±0.00
		**8**	5.69±0.00	-	5.88±0.03	-	5.61	-	-	5.66	5.68	5.75±0.00
		**15**	5.69±0.00	-	5.66±0.01	-	5.59	5.63	5.22	5.37	5.34±0.00	-
		**25**	5.69±0.00	-	5.59±0.01	-	5.32	-	5.34	-	-	-
	**0.901**	**2**	5.71±0.00	-	5.93±0.02	-	5.66	-	-	5.75	5.78	5.70±0.00
		**8**	5.71±0.00	-	5.94±0.02	-	5.63	-	-	5.66	5.67	5.64±0.00
		**15**	5.71±0.00	-	5.75±0.01	-	5.62	5.55	5.31	-	5.35	5.36±0.00
		**25**	5.71±0.00	-	5.65±0.00	-	5.32	5.26	5.19	-	-	5.41±0.00
	**0.925**	**2**	5.78±0.00	-	5.77±0.01	-	5.75	-	-	5.83	5.65	5.78±0.00
		**8**	5.78±0.00	-	5.76±0.06	-	5.73	-	-	5.53	5.47	5.54±0.00
		**15**	5.78±0.00	-	5.81±0.01	-	5.61	5.39	5.64	-	5.7	5.92±0.00
		**25**	5.78±0.00	5.68±0.12	5.49±0.00	-	5.43	5.48	5.87	-	-	-
