# Supplementary material for: Growth and Non-Thermal Inactivation of Staphylococcus aureus in Sliced Dry-Cured Ham in Relation to Water Activity, Packaging Type and Storage Temperature
Source: Foods. 2023 May 30;12(11):2199. doi: 10.3390/foods12112199 (PMC10252274; doi:10.3390/foods12112199)
Supplement: Supplementary file 1 [file foods-12-02199-s001.zip › Table S3.pdf]

Table S3. Lactic acid bacteria counts (Log_{10} cfu/g) measured in DCH with different a_w content and type of packaging stored at various storage temperatures.

Packaging	a_w	T°C	Time (days)									
			0	5	12	16	28	40	147	196	231	293
	**0.861**	15	0.00±0.00	0.00±0.00	-	-	1.85	-	-	-	-	-
		**20**	0.00±0.00	1.65	0.00	-	0.00	-	-	-	-	-
		**25**	0.00±0.00	0.70	0.00	-	0.00	-	-	-	-	-
	**0.901**	**15**	0.00±0.00	0.00	-	-	4.13	-	-	-	-	-
**Air**		**20**	0.00±0.00	-	5.14	-	-	-	-	-	-	-
		**25**	0.00±0.00	-	4.39	-	4.03	-	-	-	-	-
	**0.925**	**15**	3.12±2.57	-	5.26	-	>6.00	-	-	-	-	-
		**20**	3.12±2.57	>6.00	7.18	-	>6.00	-	-	-	-	-
		**25**	3.12±2.57	>6.00	6.87	-	>6.00	-	-	-	-	-
	**0.861**	**2**	0.00±0.00	-	0.00	0.00±0.00	0.00	-	-	0.00	0.00	0.00
		**8**	0.00±0.00	-	0.00	0.00±0.00	3.60	-	-	0.00	0.00	0.00
		**15**	0.00±0.00	-	0.00	0.00±0.00	0.00	-	-	0.00	0.00	0.00
		**20**	0.00±0.00	-	0.00	2.07±2.93	0.00	-	-	0.00	0.00	0.00
		**25**	0.00±0.00	-	0.00	1.89±2.67	0.00	-	-	0.00	0.00	0.00
	**0.901**	**2**	0.00±0.00	-	1.70	1.80±2.55	0.00	-	-	0.00	0.00	0.00
		**8**	0.00±0.00	-	2.00	0.00±0.00	0.00	-	-	0.00	4.51	0.00
**Vacuum**		**15**	0.00±0.00	-	1.00	2.10±2.97	4.91	-	-	-	4.62	5.45
		**20**	0.00±0.00	-	4.60	5.50±0.70	6.14	5.50	5.31	-	-	-
		**25**	0.00±0.00	-	0.00	4.75±0.27	3.90	6.59	0.00	-	-	-
	**0.925**	**2**	3.12±2.57	-	3.73	4.85±0.35	5.60	-	-	6.52	6.58	0.00
		**8**	3.12±2.57	-	4.14	4.30±0.00	4.20	-	-	4.34	6.70	0.00
		**15**	3.12±2.57	-	5.31	6.48±2.10	7.84	-	-	-	6.11	5.52
		**20**	3.12±2.57	-	5.93	5.24±1.28	5.38	6.76	6.08	-	-	-
		**25**	3.12±2.57	-	7.34	6.80±0.94	6.76	6.67	-	-	-	-
	**0.861**	**2**	0.00±0.00	-	0.00	-	3.30	-	-	0.00	0.00	0.00
		**8**	0.00±0.00	-	0.00	-	0.00	-	-	0.00	0.00	0.00
		**15**	0.00±0.00	-	2.56	-	4.00	0.00	0.00	0.00	0.00	-
		**25**	0.00±0.00	-	0.00	-	4.85	-	2.30	-	-	-
	**0.901**	**2**	0.00±0.00	-	0.00	-	0.00	-	-	0.00	0.00	0.00
**MAP**		**8**	0.00±0.00	-	1.70	-	4.66	-	-	0.00	0.00	6.61
		**15**	0.00±0.00	-	0.00	-	3.60	4.53	4.08	-	0.00	0.00
		**25**	0.00±0.00	-	0.00	-	4.56	4.00	0.00	-	-	0.00
	**0.925**	**2**	3.12±2.57	-	1.85	-	3.30	-	-	6.69	6.72	0.00
		**8**	3.12±2.57	-	4.66	-	4.74	-	-	6.83	6.96	6.59
		**15**	3.12±2.57	-	2.90	-	5.56	>6.00	6.87	-	6.90	6.28
		**25**	3.12±2.57	2.52±1.04	5.11	-	6.76	6.45	4.26	-	-	-
