# Supplementary material for: Growth and Non-Thermal Inactivation of Staphylococcus aureus in Sliced Dry-Cured Ham in Relation to Water Activity, Packaging Type and Storage Temperature
Source: Foods. 2023 May 30;12(11):2199. doi: 10.3390/foods12112199 (PMC10252274; doi:10.3390/foods12112199)
Supplement: Supplementary file 1 [file foods-12-02199-s001.zip › Table S4.pdf]

Table S4. Developed predictive models of *S. aureus* inactivation in dry-cured ham in terms of log reduction, $Log_{10}(N/N_0)$, as a function of storage temperature (T, in ºC) and time (t, in days) for each type of packaging system, i.e., air, vacuum and modified atmosphere.

Packaging type	Predictive model[a]
**Air**	$Log_{10}\,(N/N_0) = -((t/10^{Log_{10}(-1.848\,+\,0.263\cdot \mathrm{T}\,-0.006\cdot T^2)})^{0.495})$
**Vacuum**	$Log_{10}\,(N/N_0) = -((t/10^{Log_{10}(2.597-0.09\cdot \mathrm{T}+0.002\cdot T^2)})^{0.768})$
**MAP**	$Log_{10}\,(N/N_0) = -((t/10^{Log_{10}(3.088-0.133\cdot \mathrm{T}+0.003\cdot T^2)})^{0.838})$

[a]: for dry-cured ham (pH 5.80 ± 0.06) showing three different aw: low, medium and high, selected to cover the range of aw variability usually found (ca. 0.860 to 0.925)
