# Supplementary material for: Growth and Non-Thermal Inactivation of Staphylococcus aureus in Sliced Dry-Cured Ham in Relation to Water Activity, Packaging Type and Storage Temperature
Source: Foods. 2023 May 30;12(11):2199. doi: 10.3390/foods12112199 (PMC10252274; doi:10.3390/foods12112199)
Supplement: Supplementary file 1 [file foods-12-02199-s001.zip › Table S5.pdf]

Table S5. Comparison of Log_{10} reduction observed in published articles dealing with *S. aureus* behaviour in sliced DCH and that predicted by the predictive models developed in the present work. Agreement was considered if the difference between the observed and predicted values were within ± 0.5 Log_{10}.

Reference	Packaging/model used	a_w	T (°C)	pH	Agreement between the observed and predicted Log_{10} reduction (%)
**Untermann and Müller** [18]	Air	0.890	20	5.6	86% (6/7)
**Iacumin et al.** [35]	Vacuum	0.910	4	5.79	83% (5/6)
			10		67% (4/6)
			15		83% (5/6)
			25		67% (4/6)
**Christian et al.** [16]	MAP (70:30 % N_2:CO_2)	0.888	15	5.64-5.81	80% (8/10)
		0.890			90% (9/10)
		0.905			78% (7/9)
		0.921			78% (7/9)
		0.925			91% (10/11)
