# Supplementary figures and images for: Growth and Non-Thermal Inactivation of Staphylococcus aureus in Sliced Dry-Cured Ham in Relation to Water Activity, Packaging Type and Storage Temperature
Source: Foods. 2023 May 30;12(11):2199. doi: 10.3390/foods12112199 (PMC10252274; doi:10.3390/foods12112199)

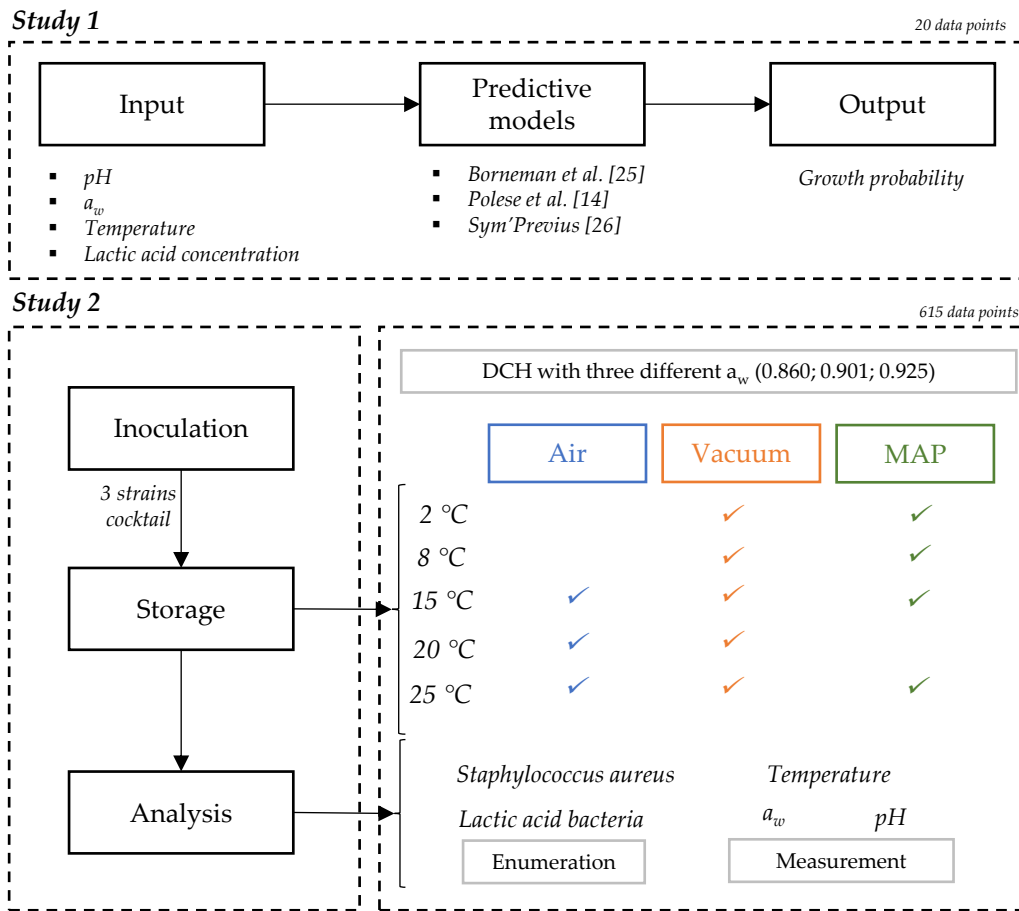

**Figure S1.** Graphical scheme of the experimental design of Study 1 and Study 2.

Supplement: Supplementary file 1 [file foods-12-02199-s001.zip › Figure S1.pdf]
